# Supplementary material for: Effects of Supplementing Tributyrin on Meat Quality Characteristics of Foreshank Muscle of Weaned Small-Tailed Han Sheep Lambs
Source: Animals (Basel). 2024 Apr 19;14(8):1235. doi: 10.3390/ani14081235 (PMC11047446; doi:10.3390/ani14081235)
Supplement: Supplementary file 1 [file animals-14-01235-s001.zip › animals-2926913-supplementary.pdf]

Table S1. The effects of tributyrin on amino acids (AAs) content in foreshank muscle of Small-Tailed Han lambs

Items	Tributyrin additions, g/kg DM basis					SEM	p-values[1]		
	0	0.5	1.0	2.0	4.0		Contrast	Linear	Quadratic
∑EAAs (g/100 g foreshank muscle)	6.61^{b}	7.82^{a}	8.13^{a}	7.84^{a}	7.92^{a}	0.220	< 0.001	< 0.001	0.706
Valine	0.93^{c}	0.98^{b}	0.97^{b}	1.05^{a}	1.00^{b}	0.014	< 0.001	< 0.001	0.006
Methionine	0.30^{b}	0.40^{a}	0.43^{a}	0.36^{ab}	0.37^{a}	0.022	< 0.001	0.170	0.399
Isoleucine	0.86^{b}	0.95^{a}	0.97^{a}	0.93^{a}	0.94^{a}	0.014	< 0.001	0.003	0.406
Leucine	1.28^{c}	1.40^{c}	1.63^{b}	1.93^{a}	1.86^{a}	0.067	< 0.001	< 0.001	0.493
Phenylalanine	0.85^{b}	1.15^{a}	1.16^{a}	0.96^{ab}	1.01^{ab}	0.065	0.004	0.492	0.487
Lysine	1.49^{b}	2.01^{a}	2.05^{a}	1.68^{ab}	1.78^{ab}	0.115	0.014	0.512	0.047
Threonine	0.89	0.91	0.92	0.90	0.93	0.015	0.188	0.183	0.440
∑NEAAs (g/100 g foreshank muscle)	9.47^{c}	10.01^{b}	10.36^{a}	10.41^{a}	10.39^{a}	0.084	< 0.001	< 0.001	0.646
Serine	0.71^{b}	0.75^{a}	0.75^{a}	0.74^{a}	0.75^{a}	0.011	0.001	0.025	0.763

Proline	0.79^{c}	0.83^{b}	0.82^{bc}	0.91^{a}	0.88^{a}	0.014	< 0.001	< 0.001	0.006
Glutamic acid	2.22^{b}	2.29^{b}	2.40^{ab}	2.55^{a}	2.50^{a}	0.061	0.002	< 0.001	0.620
Glycine	0.96	0.97	0.98	1.02	1.01	0.035	0.397	0.207	0.780
Histidine	0.55^{b}	0.68^{a}	0.72^{a}	0.64^{a}	0.71^{a}	0.028	< 0.001	0.003	0.274
Alanine	1.09	1.13	1.16	1.10	1.10	0.021	0.280	0.679	0.194
Arginine	1.14^{c}	1.27^{b}	1.37^{a}	1.22^{bc}	1.25^{b}	0.031	< 0.001	0.106	0.024
Aspartic acid	1.98^{c}	2.06^{b}	2.23^{a}	2.20^{a}	2.06^{b}	0.022	< 0.001	< 0.001	0.051
∑AAs (g/100g)	16.0^{b}	17.8^{a}	18.4^{a}	18.2^{a}	18.3^{a}	0.25	< 0.001	< 0.001	0.628
Branched-chain AAs[2] (g/100g)	3.07^{d}	3.33^{c}	3.58^{b}	3.92^{a}	3.81^{a}	0.076	< 0.001	< 0.001	0.331
Umami AAs[3] (UAAs, g/100g)	4.20^{d}	4.35^{cd}	4.52^{bc}	4.76^{a}	4.66^{ab}	0.067	< 0.001	< 0.001	0.473
Sweet AAs[4] (SAAs, g/100g)	4.46^{c}	4.60^{b}	4.66^{ab}	4.69^{a}	4.68^{a}	0.022	< 0.001	< 0.001	0.771
EAA/∑AAs	0.41^{b}	0.43^{a}	0.43^{a}	0.42^{ab}	0.43^{a}	0.006	0.003	0.130	0.812
EAA/∑EAAs	0.69^{b}	0.78^{a}	0.78^{a}	0.75^{ab}	0.76^{a}	0.021	0.003	0.137	0.855

UAA/∑AAs	0.26	0.24	0.24	0.26	0.25	0.005	0.077	0.821	0.361
SAA/∑AAs	0.27[a]	0.25[b]	0.25[b]	0.25[b]	0.25[b]	0.003	< 0.001	< 0.001	0.599

DM = dry matter; EAAs = essential amino acids; NEAAs = non-essential amino acids; SEM = standard error of the mean.

[a-d] Values within a row with no common superscripts differ significantly ($p < 0.05$).

[1] Linear, linear effect of tributyrin; Quadratic, quadratic effect of tributyrin.

[2] Branched-chain amino acids including valine, isoleucine and leucine.

[3] Umami amino acids including both glutamic acid and aspartic acid.

[4] Sweet amino acids including threonine, serine, glycine, alanine and proline.

Table S2. The effects of tributyrin on fatty acid content in foreshank muscle of Small-Tailed Han lambs

Items	Tributyrin additions, g/kg DM basis					SEM	*p*-values[1]		
	0	0.5	1.0	2.0	4.0		Contrast	Linear	Quadratic
∑*SFAs (mg/100 g foreshank muscle)*	2,010[c]	2,083[bc]	2,101[bc]	2,136[b]	2,405[a]	34.9	< 0.001	< 0.001	0.610
C4:0	4.57[bc]	4.88[b]	4.19[c]	4.58[bc]	5.50[a]	0.191	0.304	0.012	0.105
C10:0	6.78[c]	6.58[c]	7.53[ab]	7.21[bc]	8.12[a]	0.240	0.036	< 0.001	0.017
C12:0	7.12[b]	6.81[c]	7.06[bc]	7.31[b]	8.23[a]	0.10	0.044	< 0.001	0.155
C13:0	10.6[c]	12.2[c]	16.3[b]	17.3[b]	21.5[a]	0.98	< 0.001	< 0.001	0.139
C14:0	106[c]	111[c]	122[ab]	120[b]	130[a]	3.14	< 0.001	< 0.001	0.096
C15:0	14.7[d]	16.0[c]	18.2[a]	17.1[b]	18.8[a]	0.29	< 0.001	< 0.001	< 0.001
C16:0	1,043[c]	1,060[bc]	1,073[bc]	1,106[b]	1,174[a]	15.5	0.001	< 0.001	0.940
C17:0	42.0[c]	43.5[bc]	44.4[ab]	44.8[ab]	46.3[a]	0.69	0.001	< 0.001	0.803
C18:0	699[b]	741[b]	734[b]	740[b]	903[a]	30.6	0.022	< 0.001	0.760

C20:0	2.84^{b}	2.54^{b}	2.52^{b}	2.82^{b}	3.57^{a}	0.145	0.899	< 0.001	0.961
C21:0	15.1^{b}	14.4^{bc}	13.4^{c}	14.2^{bc}	17.2^{a}	0.51	0.565	0.017	0.659
C22:0	5.00^{b}	5.10^{b}	4.59^{b}	4.67^{b}	6.43^{a}	0.275	0.527	0.007	0.969
C23:0	51.4^{bc}	56.5^{ab}	53.0^{bc}	48.3^{c}	61.1^{a}	1.76	0.103	0.052	0.443
∑UFAs (mg/100 g foreshank muscle)	$2,430^{a}$	$2,121^{b}$	$2,042^{b}$	$1,819^{c}$	$1,632^{d}$	65.6	< 0.001	< 0.001	0.318
C14:1	8.74^{ab}	10.1^{a}	6.89^{b}	10.1^{a}	5.99^{b}	0.938	0.668	0.068	0.002
C15:1	17.1^{a}	10.5^{b}	11.6^{b}	12.3^{b}	7.5^{b}	1.66	< 0.001	0.002	0.829
C16:1	87.2^{a}	88.0^{a}	78.1^{b}	74.2^{b}	71.5^{b}	2.31	< 0.001	< 0.001	0.286
C17:1	38.3^{a}	32.9^{bc}	34.9^{ab}	32.9^{bc}	30.1^{c}	1.25	< 0.001	< 0.001	0.166
C20:1n9	6.27^{a}	6.29^{a}	5.30^{b}	4.43^{b}	2.57^{c}	0.309	< 0.001	< 0.001	0.391
C18:1n9t	208^{a}	197^{ab}	173^{b}	182^{b}	126^{c}	8.19	< 0.001	< 0.001	0.037
C18:1n9c	$1,774^{a}$	$1,489^{b}$	$1,478^{b}$	$1,264^{c}$	$1,169^{c}$	65.9	< 0.001	< 0.001	0.153
C18:2n6t	18.5^{a}	16.0^{ab}	11.8^{c}	14.4^{bc}	9.1^{d}	1.04	< 0.001	< 0.001	0.010

C18:2n6c	228^{a}	226^{a}	201^{ab}	185^{b}	187^{b}	9.4	0.008	< 0.001	0.748
C18:3n3	11.1^{a}	10.8^{ab}	8.53^{bc}	7.91^{c}	5.00^{d}	0.817	0.002	< 0.001	0.262
C18:3n6	4.31	3.78	5.04	6.14	3.32	0.223	0.308	0.599	0.346
C20:2	4.22	5.83	7.52	4.98	3.22	0.857	0.227	0.296	0.200
C20:3n6	5.03^{a}	4.71^{a}	2.81^{b}	1.77^{b}	1.46^{b}	0.557	< 0.001	< 0.001	0.580
C20:5n3	3.65^{a}	3.68^{a}	2.87^{a}	2.89^{a}	1.80^{b}	0.327	0.025	0.007	0.214
C24:1	6.15^{b}	9.93^{a}	8.51^{ab}	7.28^{b}	3.41^{c}	0.800	0.212	0.002	0.222
C22:6n3	8.12^{a}	6.16^{ab}	5.11^{b}	6.07^{ab}	4.42^{b}	0.724	0.001	0.002	0.346
MUFAs[2] (mg/100 g foreshank muscle)	$2,146^{a}$	$1,844^{b}$	$1,796^{b}$	$1,588^{c}$	$1,417^{c}$	64.1	< 0.001	< 0.001	0.258
PUFAs[3] (mg/100 g foreshank muscle)	283^{a}	277^{a}	245^{b}	230^{bc}	215^{c}	9.3	< 0.001	< 0.001	0.441
MUFA/SFA	1.07^{a}	0.88^{b}	0.85^{b}	0.74^{c}	0.58^{d}	0.03	< 0.001	< 0.001	0.272
PUFA/SFA	0.14^{a}	0.13^{a}	0.11^{b}	0.10^{b}	0.08^{c}	0.004	< 0.001	< 0.001	0.385
$\sum$n3 (mg/100 g foreshank muscle)	22.8^{a}	19.9^{b}	16.5^{c}	17.6^{bc}	11.2^{d}	0.93	< 0.001	< 0.001	0.034

Σn6 (mg/100 g foreshank muscle)	256[a]	251[a]	221[b]	208[b]	200[b]	9.3	< 0.001	< 0.001	0.498
n6/n3	11.8[b]	12.9[b]	13.7[b]	11.6[b]	17.8[b]	0.70	0.006	< 0.001	0.023
Atherogenicity index[4]	0.61[d]	0.72[c]	0.77[c]	0.89[b]	1.06[a]	0.031	< 0.001	< 0.001	0.617
Thrombogenicity index[5]	1.38[d]	1.64[c]	1.72[c]	1.98[b]	2.52[a]	0.070	< 0.001	< 0.001	0.676

DM = dry matter; SEM = standard error of the mean; SFAs = saturated fatty acids; UFAs = unsaturated fatty acids.

[a-d] Values within a row with no common superscripts differ significantly ($p < 0.05$).

1 Linear, linear effect of tributyrin; Quadratic, quadratic effect of tributyrin.

2 Monounsaturated fatty acids including C14:1, C15:1, C16:1, C17:1, C20:1, C18:1n9t, C18:1n9c and C24:1.

3 Polyunsaturated fatty acids including C18:2n6t, C18:2n6c, C18:3n6, C20:2, C18:3n3, C18:3n6 and C22:6n3.

4 Atherogenicity index = (12:0 + 4 × 14:0 + 16:0)/ (MUFA + PUFA) calculated according to Ulbricht and Southgate (1991).

5 Thrombogenicity index = (12:0 + 16:0 + 18:0)/ [(0.5 × MUFA) + (0.5 × n-6 PUFA) + (3 × n-3 PUFA) + (n-3 PUFA/n-6 PUFA)] calculated according to Ulbricht and Southgate (1991).
